# 10543 Assessing Sexual Health Services at a public university in the Deep South

**DOI:** 10.1017/cts.2021.472

**Published:** 2021-03-30

**Authors:** Andres Camino, Meghan Whitfield, Nicholas Van Wagoner

**Affiliations:** University of Alabama at Birmingham School of Medicine

## Abstract

ABSTRACT IMPACT: Our work helps show universities that embedding dedicated sexual health clinics within university health and wellness clinics may expand the amount of students they see for sexual health screenings during a time of increased sexual behavior and exploration. OBJECTIVES/GOALS: The National College Health Association reports that college students have frequent, condomless sex. Student health and wellness clinics (SHWC) offer sexual health services, but few have dedicated sexual health clinics (SHC). We evaluated sexual health service use at a university SHWC after implementation of a dedicated SHC two half-days per week. METHODS/STUDY POPULATION: This was a retrospective analysis of data collected from patients receiving sexual health screening at the University of Alabama at Birmingham (UAB) SHWC between January 2015 and June 2019. Demographic variables, sexual behaviors, reason for testing, and rates of STIs were extracted from the electronic medical record and were compared by clinic (SHC vs. SHWC). Data on screening visits of patients over 18 were included in the final analysis. Variables were summarized with frequencies and percentages. Univariate models were fit, and multi-variable models will be fit, selecting variables with p values of 0.1 or less. Odds ratios with corresponding 95% confidence intervals for univariate analysis are presented. The study was approved by the UAB Institutional Review Board. RESULTS/ANTICIPATED RESULTS: A total of 5025 STI screenings were performed. Males (OR 4.13; 3.61-4.72), undergraduates (OR 1.33; 1.15-1.54), and persons reporting sex with the same sex (OR 1.88; 1.56-2.28), were significantly more likely to seek care at the SHC. Students with symptoms were more likely to seek care at the SHWC (OR 0.53; 0.47-0.61), while persons who reported contact with STIs were more likely to seek care at the SHC (OR 2.88; 2.22-3.74). The overall percentage of positive screenings was 9.3% for chlamydia (CT), 3.0% for gonorrhea (GC), 0.8% for trichomoniasis (TV), 0.7% for syphilis, and 0.3% for HIV with higher percentages of positive for CT (OR 1.60; 1.30-1.96) and GC (OR 2.02; 1.44-2.85) in the SHC. A greater percentage of positives for TV (OR 0.37; 0.14-0.96) was found in the SHWC. DISCUSSION/SIGNIFICANCE OF FINDINGS: Based on demographics of persons utilizing services, embedding a dedicated SHC within a university SHWC may expand populations reached for STI screening. With higher percentages of patients testing positive for CT and GC, a SHC may allow for greater diagnosis and treatment of STIs in general screening and persons presenting as contacts.

